# Stroke-Like Migraine Attacks After Radiation Therapy (SMART) Syndrome: A Case Report

**DOI:** 10.7759/cureus.63599

**Published:** 2024-07-01

**Authors:** Ashraf V Valappil, Danish Ahammed PK, Sellam Karunanidhi, Harish Babu SP, Sujith Janardhanan

**Affiliations:** 1 Neurology, Aster Malabar Institute of Medical Sciences Ltd (MIMS) Hospital Calicut, Kozhikode, IND; 2 Nuclear Medicine, Aster Malabar Institute of Medical Sciences Ltd (MIMS) Hospital Calicut, Kozhikode, IND; 3 Radiology, Aster Malabar Institute of Medical Sciences Ltd (MIMS) Hospital Calicut, Kozhikode, IND

**Keywords:** stroke-like migraine attacks after radiotherapy (smart), seizures, fdg pet scan, magnetic resonance imaging (mri), delayed radiation induced neurotoxicity

## Abstract

Stroke-like migraine attacks after radiation therapy (SMART) syndrome is a rare and delayed complication of brain irradiation involving impaired cerebrovascular autoregulation, and diagnosis is based on distinct clinic-radiographic findings and exclusion of differentials. We report a 38-year-old man, who received cranial irradiation 28 years before and developed episodes of headache and visual aura, followed by left hemianopia, aphasia, behavioral disturbances, and focal seizures. An MRI of the brain revealed gyral swelling with restricted diffusion and mild contrast enhancement over the right temporoparietal and occipital region, and fludeoxyglucose-FDG PET scan showed hyperperfusion in the corresponding brain region. He improved completely with pulse steroids and antiseizure medications. The recognition of this syndrome is important as we can reassure patients and their families and help avoid unnecessary and invasive diagnostic tests.

## Introduction

Radiation therapy plays a major role in the management of malignancies affecting the brain and is an additional treatment modality to neurosurgery and chemotherapy. Cranial irradiation can cause acute or delayed neurotoxicity. Stroke-like migraine attacks after radiation therapy (SMART) syndrome is a rare delayed neurological complication of cranial radiotherapy observed in both adults and children treated with cranial radiation therapy [1]. The onset of SMART syndrome can be delayed up to 37 years after radiation [2]. Patients with SMART syndrome usually present with stroke-like symptoms, including visual disturbances, speech impairment, facial weakness, hemiparesis, migraine-like headaches, and seizures [2,3]. MRI typically shows unilateral gyral swelling and gadolinium enhancement, not confined to vascular territories, sparing white matter in the previously irradiated field [4]. We report a young man with clinical features of SMART syndrome with typical MRI features and interesting fludeoxyglucose-PET findings who received cranial radiotherapy about 25 years ago.

## Case presentation

A 38-year-old left-handed male presented with a history of headaches, hemianopia, seizures, and aphasia. At the age of 13 years, he was diagnosed with right cerebellar space-occupying lesion, probably astrocytoma. He underwent excision surgery, followed by radiation therapy. At the age of 20 years, he was diagnosed with Type 1 diabetes mellitus and has been on insulin since then at 32 years of age, he was detected to have hypothyroidism, and he was started on thyroid hormone replacement. Four years ago, he started getting episodic, hemicranial headaches with visual aura. Each episode lasted for a few hours, and frequency and severity got worse for the past two months. Forty days before his presentation to us, he developed a severe headache and blurring of vision on his left side. He was hospitalized at another center. His MRI brain there reportedly revealed an infarct in the right parieto-occipital region, and he was put on antiplatelets. After a few days of hospitalization, he started getting episodes of visual illusions on the left visual field associated with behavioral arrest, and he was given sodium valproate, considering the possibility of seizures. After discharge, the patient continued to have severe right-sided headaches, and the blurring of vision persisted. Two weeks before his presentation to us, he suddenly became confused and could not understand his relatives' speech or convey his thoughts as speech to relatives. His speech was irrelevant to the situation, and he repeated the sentences without any purpose. Two days before presentation to us, the patient had two episodes of focal seizures in the form of recurrent facial twitching with impaired awareness lasting for one to two minutes, followed by drowsiness.

On examination, his vitals, including blood pressure, were normal. He was confused, his comprehension impaired, with normal fluency suggestive of Wernicke’s aphasia. He had left hemianopia, and other cranial nerves were normal. His limb power, coordination, and sensory system examination were normal.

**Figure 1:**
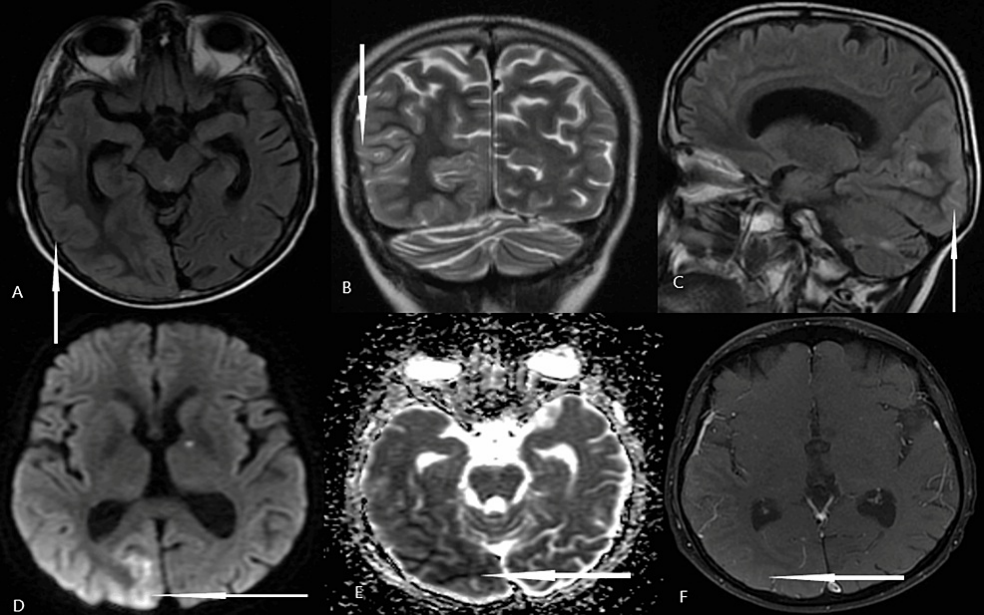
MRI brain images MRI brain: top panel (A) T2 FLAIR axial, (B) T2-weighted coronal (C) T2 FLAIR sagittal, showing cortical gyral edema involving right parieto-occipital area Bottom panel: (D) diffusion-weighted sequence showing diffusion restriction in the corresponding area, (E) apparent diffusion coefficient (ADC) sequence showing a drop in ADC value, and (F) T1 post-gadolinium sequence showing faint enhancement

Routine blood investigations including complete blood count, renal function tests, liver function tests, and electrolytes were unremarkable. Blood sugar was 186 mg/dL, and HbA1c was 7. An MRI brain with gadolinium contrast was done, which showed right temporoparietal and occipital gyral swelling with mild contrast enhancement with diffusion hyperintensity (Figure 1). Additionally, an acute lacunar infarct was noted in the left anterior thalamus. Bilateral cerebellar gliosis (postoperative changes) was also noted. Differential diagnoses based on clinical and imaging findings were considered as follows: (a) central nervous system vasculitis, (b) autoimmune encephalitis, (c) Creutzfeldt-Jakob Disease (CJD), and (d) SMART syndrome.

**Figure 2:**
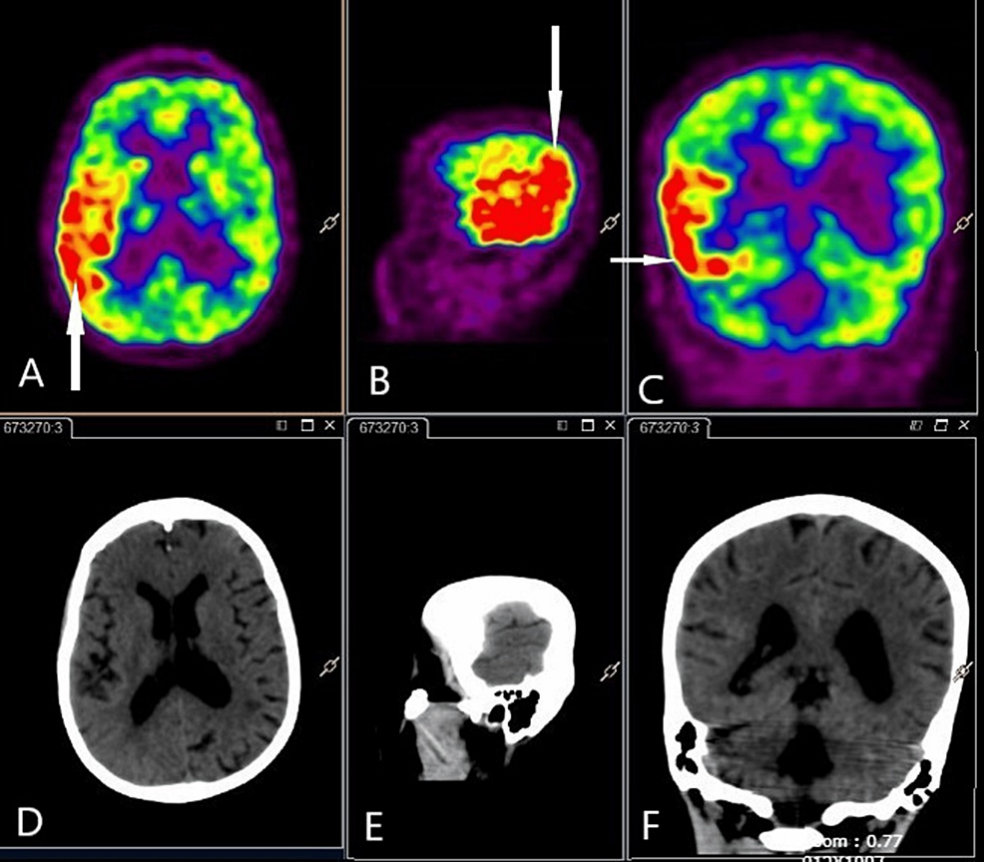
Fludeoxyglucose-PET CT images Fludeoxyglucose-PET CT images (A) axial, (B) sagittal, (C) coronal, sequences showing hypermetabolism, as shown by arrows over the right parieto-occipital region The bottom panel (D, E, F) shows the corresponding plain CT images

Workups for systemic vasculitis, such as antinuclear antibody, rheumatoid factor, cytoplasmic and perinuclear anti-neutrophil cytoplasmic antibodies (c- and p-ANCA, respectively), hepatitis C virus (HCV), HBsAg, and anticardiolipin antibodies, were all negative. A cerebral digital subtraction angiography (DSA) also did not show any evidence of vasculitis. Cerebrospinal fluid analysis was normal except for mild elevation of protein. CSF autoimmune encephalitis panel and viral encephalitis panel were negative. CSF 14-3-3 levels done to rule out CJD were negative. The EEG done did not show any periodic complexes characteristic of CJD, but there were recurrent electrographic seizures over the right parieto-occipital and temporal region observed in the EEG. FDG PET scan showed only hypermetabolism over the right parieto-occipital area (Figure 2).

In summary, this patient presented with recurrent transient neurological deficits attributable to unilateral cortical involvement in the form of aphasia, hemianopia, facial palsy, and focal seizures, associated with antecedent history of recurrent headache attacks. Considering the above clinical features with the radiological finding of unilateral parieto-occipital contrast-enhancing gyral swelling in the background of a remote history of cranial irradiation for a posterior fossa tumor, we arrived at a provisional diagnosis of SMART syndrome after ruling out all other possible differential diagnoses. The patient was managed with pulse steroids (injection of methylprednisolone 500 mg once daily for five days), antiseizure medications, antiplatelets, statins, antihypertensives, and antidiabetic medications. The patient had significant clinical improvement in mentation, and speech comprehension, and had no further seizures. He completely recovered at six months follow-up.

## Discussion

Cranial irradiation can cause various delayed neurological complications, and these include SMART syndrome, peri-ictal pseudoprogression (PIPG), acute late-onset encephalopathy after radiation therapy (ALERT) syndrome, focal cerebral radiation necrosis, and short-lasting focal deficits (SLFD) [5]. Various features of these syndromes are discussed in Table 1. SMART syndrome can affect any age group and has a male predominance. The mean age of onset of SMART syndrome is around 45 years, ranging from 3.5 to 88 years of age [1]. The time to symptom onset after radiation therapy ranges from one to 37 years, with an average of 20 years [2,6]. The SMART syndrome is reported to occur more commonly in patients with primary brain tumors, and these tumors more commonly affect the posterior part of the brain (i.e., parietal occipital-temporal lobes, cerebellum, and brainstem), as in our patient [1,2].

**Table 1:** Features of different delayed radiation-induced neurological syndromes Adapted and modified from Maramattom et al. [5] ALERT: acute late-onset encephalopathy after radiation therapy; PIPG: peri-ictal pseudoprogression; SMART: stroke-like migraine attacks after radiation therapy

Presentation	SMART syndrome	PIPG	ALERT	Focal cerebral radiation necrosis
Clinical features	Stroke-like events, migrainous headaches, seizures	Partial seizures predominant	Focal neurological deficits, altered mental status	Partial seizures, encephalopathy, focal deficits
MRI findings	Diffuse unilateral cortical thickening with a gadolinium enhancement of the cerebral gyri, especially over the parieto-occipital cortex, Leptomeningeal enhancement rare	Focal cortical and/ or leptomeningeal enhancing lesions Superficial lesions	Unilateral or bilateral multiple subcortical patchy enhancing lesion	The new enhancing lesion, enlargement of a previously enhancing lesion, or worsening signal abnormality or enhancement after radiation therapy subcortical white matter edema may be prominent. The absence of restricted diffusion and reduced relative cerebral blood volume favors necrosis over tumor recurrence
Treatment	Steroids, anticonvulsants, Verapamil	Steroids, anticonvulsants	Steroids, anticonvulsants	Steroids

The pathophysiology of SMART syndrome is currently unclear and has been postulated as multifactorial. One of the most popular hypotheses is postradiation inflammatory endothelial damage that preferentially targets small vessels as a possible mechanism [7]. Radiation-induced neuronal dysfunction is another important hypothesis. Neuronal dysfunction can cause an impaired trigeminovascular system or reduce the threshold of cortical spreading depression [2]. One more mechanism proposed is radiation-induced mitochondrial dysfunction [7,8].

Patients commonly present with headaches, which are often severe, throbbing, and unilateral, and can be associated with nausea, vomiting, and photophobia with or without the presence of an aura [9]. Patients also develop focal neurological deficits, which are considered "stroke-like," including aphasia, visual impairment, hemiparesis, and hemisensory changes, and can repeatedly occur [9]. Although not notable in the name, seizure is a common clinical manifestation of SMART syndrome. One review of published case series reported a seizure prevalence of up to 60% [10]. Seizures can be focal or generalized and may be lethal, warranting rapid control with antiseizure medications. Many patients can have subclinical seizures, and some of the negative neurological symptoms such as aphasia, hemianopia, and hemiparesis can occur because of seizures [11]. Our patient had recurrent focal electrographic seizures.

MRI plays a crucial role in the diagnosis of SMART syndrome. In SMART syndrome, typical MRI features are unilateral, reversible, cortical swelling appreciated on MRI T2-weighted and fluid-attenuated inversion recovery (FLAIR) imaging with corresponding gyriform enhancement in a distribution inconsistent with vascular territories. Our patient had gyral swelling, but gadolinium enhancement was not marked as described in most of the published case reports. There is a preferential involvement of the posterior part of the brain, such as temporal, occipital, and parietal, typically sparing the subcortical and deep white matter [12]. Similar findings can be seen in status epilepticus (SE), ictal, or postictal phase. One important differentiating feature is probably cerebral localization; SE has a predilection for the limbic system and can be multifocal, whereas SMART syndrome mainly affects the cerebral grey matter with a predilection for the posterior cortex [12,13]. MRI findings in SMART syndrome are usually transient, except in a few patients who may have permanent findings because of cortical laminar necrosis [13]. Our patient had a lacunar infarct because of small vessel disease. Delayed vasculopathy associated with prior radiation is known to cause stroke. This can be a result of large vessel stenosis or small vessel disease. A decrease in N-acetyl-aspartate levels and increased levels of choline and creatine was found in magnetic resonance (MR) spectroscopy in a patient with SMART syndrome [14]. There are few reports discussing fludeoxyglucose-PET (FDG-PET) findings in patients with SMART syndrome. The F-18 FDG PET/CT is reported to show cerebral cortical hypermetabolism involving the area overlying the enhancing region on T1 post-contrast imaging [15]. Whether it is because of active seizures or SMART syndrome itself is still unclear. Our patient had hypermetabolism in FDG-PET in the acute phase. The PET scan also helps exclude a potential neoplastic recurrence in the brain, and it is a diagnostic tool for whole-body oncology exploration for these patients with a history of neoplasm.

Although there are no established criteria for SMART syndrome, a diagnostic criterion has been proposed by Black et al. [16], which includes defining clinical and radiographic findings as shown below.

Diagnostic Criteria for SMART Syndrome [14]

• Remote history of external beam cranial irradiation without evidence of residual or recurrent neoplasm

• Prolonged and reversible signs/symptoms that are referable to a unilateral cortical region beginning years post-radiation and may include the following: headache with the attacks, antecedent migraine with or without aura starting after irradiation, confusion, seizures, aphasia, visuospatial deficits, hemisensory deficits, hemiparesis

• Transient, diffuse, and unilateral cortical enhancement with gadolinium that involves the cerebral gyri while sparing the white matter within a previous radiation field

• Not attributed to another disorder

Our patient had all the points described in the above diagnostic criteria. Gadolinium enhancement in MRI is not marked as mentioned in the criteria.

There are currently no clear guidelines on effective treatment approaches for SMART syndrome because of the paucity of reported cases. Commonly used medications include antiseizure medications, steroids, and antihypertensive medications. Pulse steroid therapy can hasten recovery and improve MRI abnormalities [7]. The prognosis in SMART syndrome is generally good in most of the reported cases, although some patients experience incomplete recovery or recurrent episodes.

## Conclusions

Although rare, SMART syndrome is an important clinical entity to be aware of in patients who have received cranial irradiation. Symptom onset can be delayed for many years after cranial irradiation. The diagnosis of SMART syndrome requires the integration of clinical features, EEG, CSF, and neuroimaging findings. Common clinical features include stroke-like focal neurological deficits, migraine headaches, and seizures. MRI plays a major role in the diagnosis of SMART syndrome. It is important to exclude other close differential diagnoses. Early diagnosis of this syndrome is of value owing to its reversibility, which is important to explain to patients and caregivers to avoid unnecessary invasive investigations such as brain biopsy.
